# P-816. Diagnostic Performance of Molecular Sample-to-Answer Tests for Bloodstream Infections in the Clinical Setting: a Systematic Literature Review and Meta-Analysis

**DOI:** 10.1093/ofid/ofae631.1008

**Published:** 2025-01-29

**Authors:** Joan-Miquel Balada-Llasat, Yu Wang, Kristina Lindsley, Tammy C Bleak, Sarah Jiudice, Jenny Uyei, Yi Wang, Tristan T Timbrook

**Affiliations:** The Ohio State University Wexner Medical Center, Columbus, OH; IQVIA, Inc, Orchard Park, New York; IQVIA, Inc, Orchard Park, New York; bioMerieux, Salt Lake City, Utah; bioMerieux, Inc, Durham, North Carolina; IQVIA, Inc, Orchard Park, New York; IQVIA, Inc, Orchard Park, New York; bioMerieux, Salt Lake City, Utah

## Abstract

**Background:**

Rapid identification of bloodstream pathogens and antimicrobial resistance (AMR) genes by molecular tests from positive blood cultures (PBCs) have the potential to improve patient management. A systematic review and meta-analysis was conducted to evaluate diagnostic test accuracy (DTA) of molecular tests compared to phenotypic methods for detecting pathogens and AMR genes in the clinical setting.

**Figure 1.**
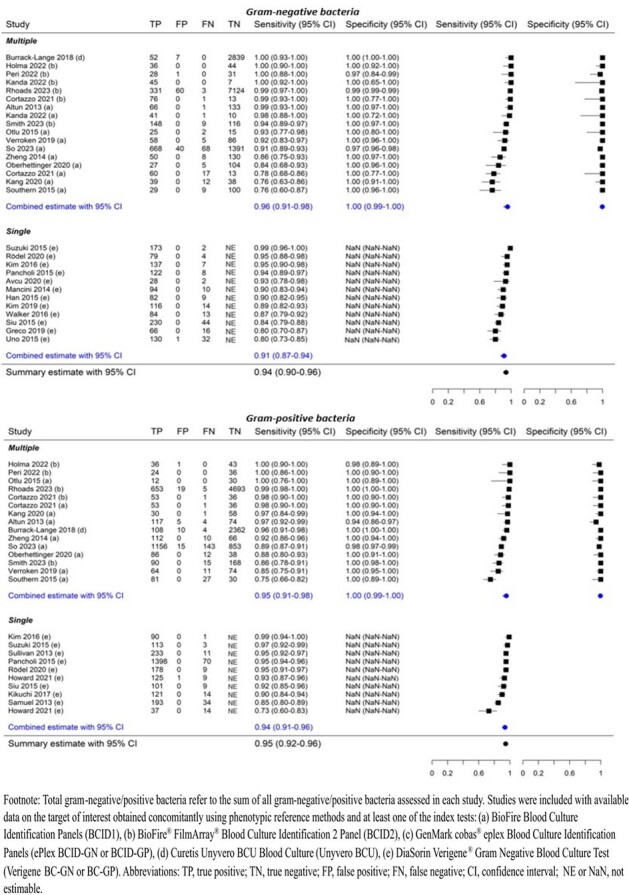
Summary sensitivity and specificity for total gram-negative bacteria and gram-positive bacteria, with subgroup by multiple and single test panel coverage.

**Methods:**

MEDLINE, EMBASE, and Cochrane databases were searched from inception to October 25, 2023. Search of conference proceedings and forward and backward searching were also done. Studies evaluating DTA of commercially available molecular sample-to-answer tests versus phenotypic methods (reference standard) in adults and children with PBCs were eligible. Risk of bias (RoB) was assessed using QUADAS-2. Summary DTA outcomes were estimated using bivariate random-effects model for gram-negative bacteria (GNB), gram-positive bacteria (GPB), yeast, gram-negative (GN) AMR, gram-positive (GP) AMR, and targets within these groups when reported by ≥ 2 studies (PROSPERO CRD42023488057).

**Figure 2.**
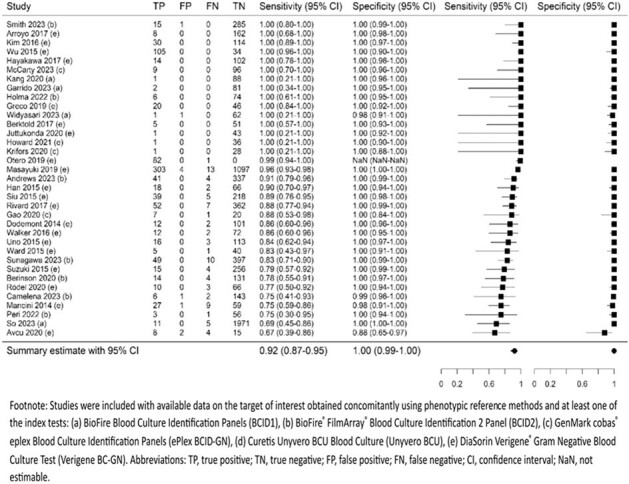
Summary sensitivity and specificity for antimicrobial resistance detected in gram-negative bacteria

**Results:**

Seventy-four studies including 24,634 samples were analyzed, most of which showed low RoB or applicability concerns. Compared with phenotypic methods, molecular tests demonstrated high DTA across all target groups, with sensitivity ≥ 92% and PPV ≥ 99% for total GNB (N=43 studies), GPB (N=38), yeast (N=24), GN AMR (N=35), and GP AMR (N=39). Specificity and NPV were 100% and 98% for GNB and 100% and 97% for GPB, respectively, among molecular tests targeting both groups (not estimable for molecular tests targeting GNB only or GPB only as true negatives are not applicable). Specificity and NPV were ≥ 99% for other target groups. High DTA was also observed for individual pathogen targets (≥ 93% sensitivity, 100% specificity, ≥ 96% PPV, and ≥ 99% NPV). Five of 7 AMR genes had high sensitivity (91%-99%) and specificity (≥ 99%). Sensitivity was lower for 2 carbapenemase genes IMP (N=4; 69%, 95% CI 15%–96%) and VIM (N=4; 70%, 95% CI 38%–90%), of which detection was missed in *P. aeruginosa*.

**Figure 3.**
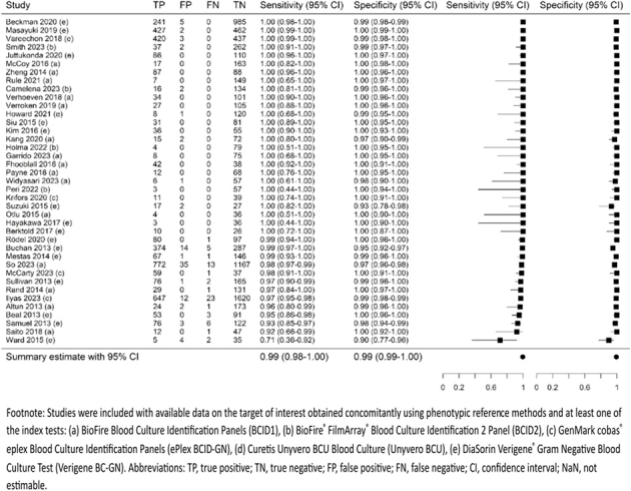
Summary sensitivity and specificity for antimicrobial resistance detected in gram-positive bacteria

**Conclusion:**

Results show excellent DTA of molecular sample-to-answer tests compared to traditional culture in identifying a broad panel of pathogens and detecting AMR in GNB and GPB.

**Table 1.**
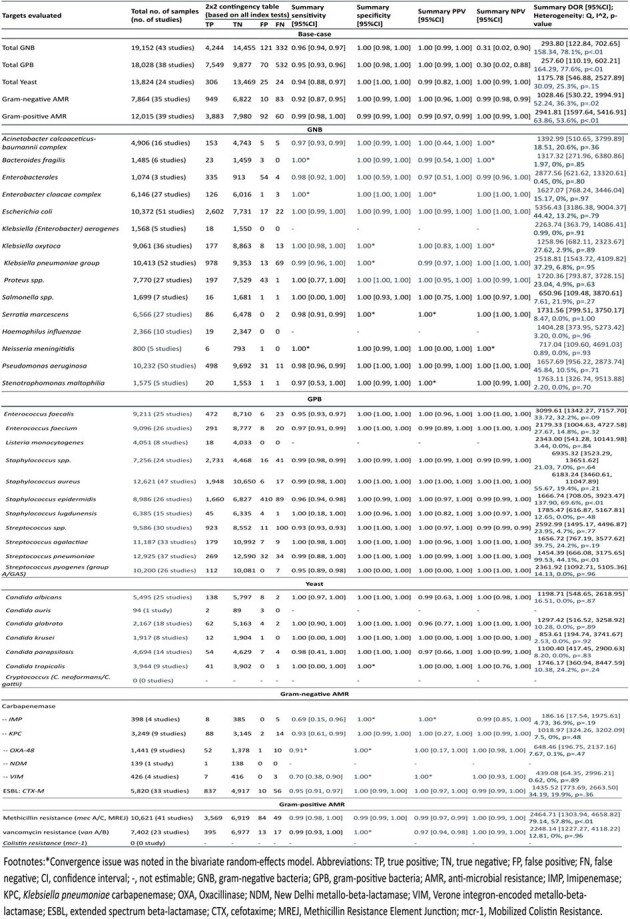
Summary of diagnostic test accuracy outcomes for targets evaluated across studies

**Disclosures:**

**Joan-Miquel Balada-Llasat, PharmD/PhD**, bioMerieux: Advisor/Consultant|bioMerieux: Grant/Research Support **Tammy C. Bleak, PharmD, MSc**, bioMerieux, Inc: employee **Sarah Jiudice, MPH**, bioMerieux, Inc: employee **Tristan T. Timbrook, PharmD**, bioMérieux: Stocks/Bonds (Public Company)

